# Changes in Cx43 and AQP4 Proteins, and the Capture of 3 kDa Dextran in Subpial Astrocytes of the Rat Medial Prefrontal Cortex after Both Sham Surgery and Sciatic Nerve Injury

**DOI:** 10.3390/ijms252010989

**Published:** 2024-10-12

**Authors:** Karolína Bretová, Viktorie Svobodová, Petr Dubový

**Affiliations:** Cellular and Molecular Neurobiology Research Group, Department of Anatomy, Faculty of Medicine, Masaryk University, CZ-62500 Brno, Czech Republic; karolina.bretova@med.muni.cz (K.B.);

**Keywords:** astrocytes, reactivity, nerve injury, tissue injury, in situ proteomics, image analysis, gap junction, aquaporins, fluoro-ruby

## Abstract

A subpopulation of astrocytes on the brain’s surface, known as subpial astrocytes, constitutes the “glia limitans superficialis” (GLS), which is an interface between the brain parenchyma and the cerebrospinal fluid (CSF) in the subpial space. Changes in connexin-43 (Cx43) and aquaporin-4 (AQP4) proteins in subpial astrocytes were examined in the medial prefrontal cortex at postoperative day 1, 3, 7, 14, and 21 after sham operation and sciatic nerve compression (SNC). In addition, we tested the altered uptake of TRITC-conjugated 3 kDa dextran by reactive subpial astrocytes. Cellular immunofluorescence (IF) detection and image analysis were used to examine changes in Cx43 and AQP4 protein levels, as well as TRITC-conjugated 3 kDa dextran, in subpial astrocytes. The intensity of Cx43-IF was significantly increased, but AQP4-IF decreased in subpial astrocytes of sham- and SNC-operated rats during all survival periods compared to naïve controls. Similarly, the uptake of 3 kDa dextran in the GLS was reduced following both sham and SNC operations. The results suggest that both sciatic nerve injury and peripheral tissue injury alone can induce changes in subpial astrocytes related to the spread of their reactivity across the cortical surface mediated by increased amounts of gap junctions. At the same time, water transport and solute uptake were impaired in subpial astrocytes.

## 1. Introduction

Astrocytes, the most abundant glial cells in the central nervous system (CNS), play a pivotal role in the cellular responses of the brain to injury [1]. Astrocytes exhibit a wide range of subpopulations within the normal adult CNS. This variability extends to neurological disease and post-CNS injury, which determines their functional implications and reactivity, regulated by specific signaling molecules [2,3]. A specific type of astrocytes, their endfeet, or cytoplasmic processes, form a boundary that separates the CNS parenchyma from non-nervous tissue. This boundary, known as the glia limitans, serves as a component of the blood–brain barrier (BBB), forming a lining for blood vessels (*glia limitans perivascularis*, GLP) and covering the CNS surface as subpial astrocytes (*glia limitans superficialis*, GLS) [4,5]. In rodents, the GLS is composed of astrocyte bodies and their cytoplasmic processes, which are connected by gap junctions, forming a syncytial sheet that covers the brain surface [6,7]. This sheet is an interface between the cortical parenchyma and the cerebrospinal fluid (CSF) in the subpial space. The pia mater, which contains fenestrations, allows the diffusion of the CSF and its components from the subarachnoid space into the subpial space at the surface of the CNS parenchyma. Consequently, the GLS functions as a structural boundary, regulating the bidirectional exchange of water and solutes between the CSF in the subpial space and the CNS parenchyma [8,9].

Astrocytic gap junctions consist primarily of connexin (Cx) 30 and 43 (Cx43), with Cx43 playing a major role in their reactivity and immunophenotyping [10,11]. These gap junctions allow the passage of water, dissolved ions, and small molecules [12,13]. Aquaporins, a family of water channel proteins, are expressed in various cell types, including those of the nervous system. Aquaporin-4 (AQP4) is the most abundant aquaporin in primate and rodent brains, found particularly in astrocytes and their cytoplasmic processes, especially endfeet forming the GLP [14,15]. Four AQP4 molecules assemble into channels that facilitate the movement of water and solutes in astrocytes [16]. High levels of Cx43 and AQP4 proteins have been observed in the astrocytic endfeet of the GLP, which are strategically positioned for the exchange between the CSF and parenchymal interstitial fluid [14,15]. Changes in Cx43 and AQP4 in the GLP have been studied after various forms of neural tissue injury [17,18].

Experimental evidence has shown that peripheral nerve injury results in the disruption of the BBB [19] and the propagation of neuroinflammatory responses within the CNS [20,21]. Additionally, peripheral nerve compression has been observed to induce cellular changes in the choroid plexus, leading to a weakening of the blood–cerebrospinal fluid barrier [22]. These experimental findings might be explained by Wallerian degeneration occurring distal to peripheral nerve injury, which serves as a source of molecular signals capable of affecting different types of CNS barriers.

The medial prefrontal cortex (mPFC) is involved in the development of co-morbid affective behavioral changes following nerve injury [23,24,25]. In our previous experiments, we found the reactivation of subpial astrocytes in the mPFC in response to sciatic nerve lesions [26]. Astrocytes and their cytoplasmic processes, which form the GLS, are interconnected by gap junctions [27] with dominant expression of Cx43, which can be visualized by immunostaining [11]. Subpial astrocytes have also been shown to exhibit AQP4 immunoreactivity [28,29]. However, quantitative changes in Cx43 and AQP4 in response to peripheral nerve injury have not yet been published.

Therefore, our current experiments were designed to investigate changes in Cx43 and AQP4 proteins in subpial astrocytes covering the mPFC at different survival times after sham operation and sciatic nerve compression (SNC). To distinguish subpial from parenchymal astrocytes, we used immunofluorescence detection with image analysis to quantify changes in Cx43 and AQP4 protein levels in subpial astrocytes of the rat mPFC. AQP4 is responsible for the uptake of solutes into the astrocytes [30]. Therefore, based on the knowledge that solutes present in blood can pass through attenuated brain barriers into the CSF in the subpial space following a peripheral nerve injury, we tested if the polar molecular tracer, TRITC-conjugated 3 kDa dextran, is taken up by reactive subpial astrocytes.

## 2. Results

### 2.1. Cx43 Immunostaining in Subpial Astrocytes

**Figure 1:**
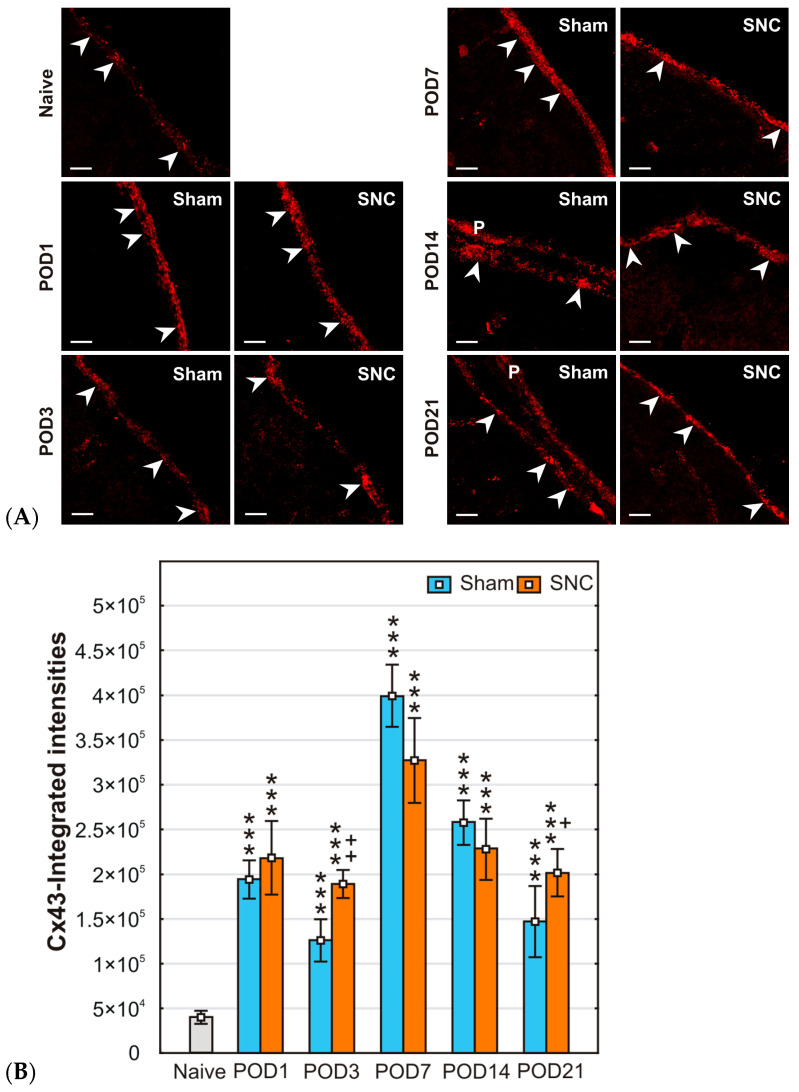
(**A**) Representative maximum projection images obtained from six Z-stack images, illustrating the immunodetection of Cx43 in the GLS of the mPFC of naïve rat and after sham or SNC operations. Arrowheads indicate GLS position, and P indicates pia mater. Scale bars = 100 μm. (**B**) The integrated intensity of Cx43-IF was significantly increased in subpial astrocytes of both sham- and SNC-operated rats during all survival periods compared to the GLS of naïve control. Results are expressed as mean ± SD. *** *p* < 0.001 compared to naïve animals; ++ *p* < 0.005, + *p* < 0.05 compared to sham operation, (*n* = 3, for each group).

Immunofluorescence staining for Cx43 revealed punctate patterns densely distributed in both the GLS profiles and, when present, in the pia mater. Representative pictures illustrating Cx43 immunofluorescence staining in the GLS on the mPFC surface of naïve, sham-, and SNC-operated rats for all survival periods examined are shown in Figure 1A.

**Figure 2:**
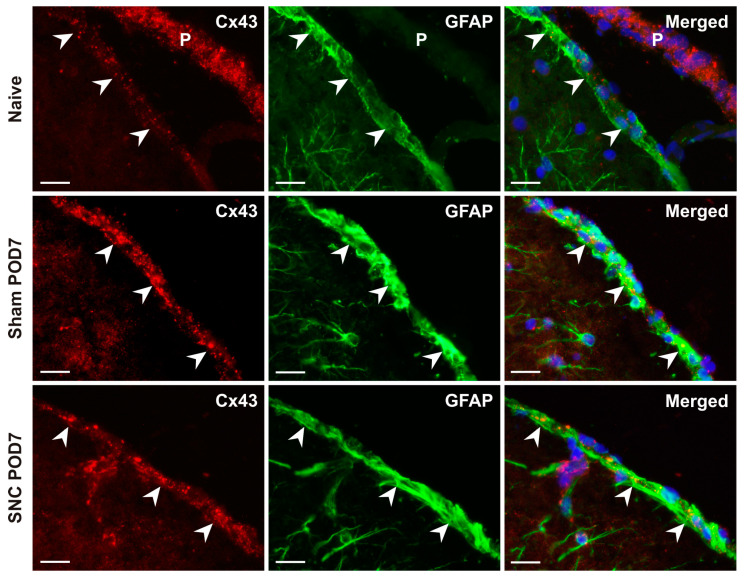
Representative pictures illustrate double immunostaining with Cx43 and GFAP antibodies, demonstrating the presence of gap junctions in the GLS formed by subpial astrocytes and their cytoplasmic processes. Merged pictures were created by overlaying the red, green, and blue fluorescence channels, with the blue color indicating the position of the cell nuclei. Arrowheads indicate GLS position, and P indicates pia mater. P. Scale bars = 100 μm.

The integrated intensity of pixels indicating Cx43 immunofluorescence showed a significant increase in subpial astrocytes from both sham- and SNC-operated rats at all survival times compared with the values from naive animals (Figure 1B). There was no statistically significant difference in GLS between sham- and SNC-operated rats at POD1, POD7, and POD14, whereas such differences were found at POD3 and POD21. The detection of a high density of Cx43-immunopositive dots in subpial astrocytes was confirmed in double immunostained sections using Cx43 and GFAP antibodies (Figure 2).

### 2.2. AQP4 Immunostaining in Subpial Astrocytes

**Figure 3:**
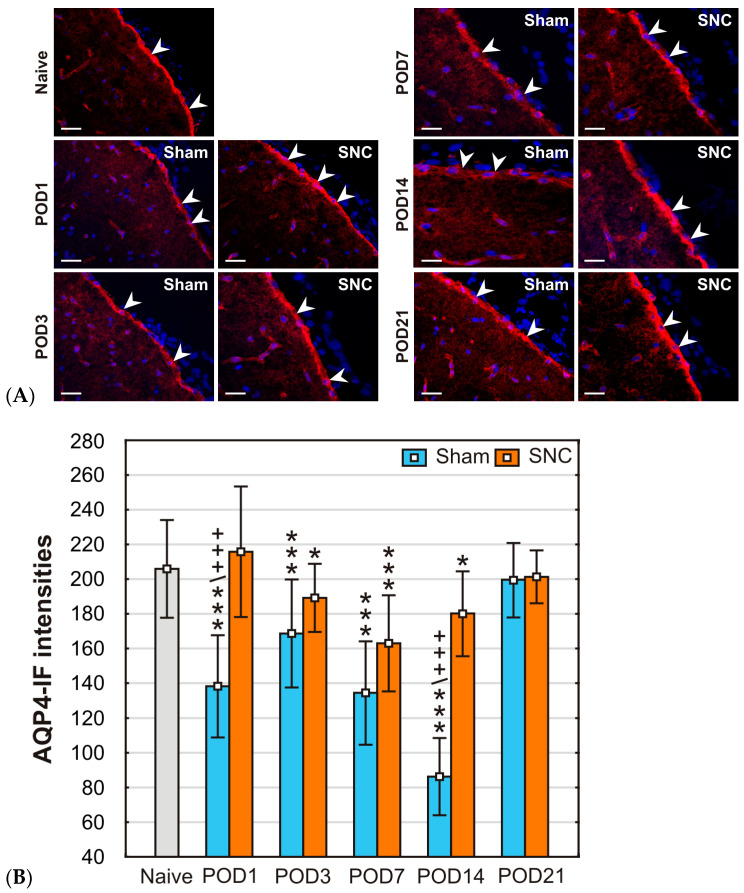
(**A**) Representative pictures illustrate AQP4-IF in the GLS of the mPFC from a naïve rat and after sham or SNC operations. Arrowheads indicate GLS position. Scale bars = 100 μm. (**B**) The dynamics of AQP4-IF intensities in subpial astrocytes following various survival periods after sham and SNC operations. Results are expressed as mean ± SD. *** *p* < 0.001 vs naïve animals; * *p* < 0.05 compared to naïve animals; +++ *p* < 0.001 compared to SNC, (n = 3, for each group).

Distinct AQP4 immunofluorescence was observed in the GLS on the surface of coronal sections of mPFC from all experimental rats. In addition, AQP4 immunostaining delineated the position of blood vessels within the mPFC parenchyma (Figure 3A).

**Figure 4:**
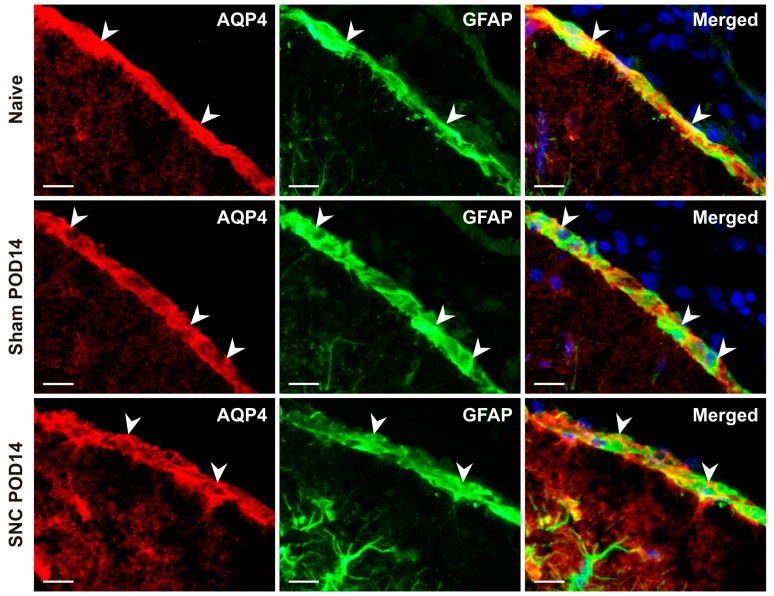
Representative images illustrate double immunofluorescence staining with AQP4 and GFAP antibodies. Merged pictures confirm the dominant presence of AQP4 protein in subpial astrocytes of the GLS. The blue color fluorescence in merged pictures indicates the position of the cell nuclei. Arrowheads indicate GLS position. Scale bars = 100 μm.

The mean intensities of AQP4-IF were significantly lower in the GLS profiles of sections obtained from the mPFC of sham-operated rats that survived for almost all time periods studied compared to those of naïve controls, except for POD21. Similarly, statistically significant reductions in AQP4-IF intensities were detected in the GLS of SNC-operated rats at POD3, POD7, and POD14 compared to the naïve control. However, the GLS of SNC-operated rats at POD1 and POD21 maintained mean intensities similar to those of naïve rats. When comparing the mean AQP4-IF intensities of the GLS in sham- and SNC-operated rats, lower values were detected after the sham operation on POD1, POD3, POD7, and POD14, but statistically significant differences were found only on POD1 and POD14 (Figure 3B). The localization of AQP4 protein predominantly in subpial astrocytes was confirmed by double immunofluorescence staining with AQP4 and GFAP antibodies (Figure 4).

### 2.3. Results of 3 kDa Dextran Uptake in Subpial Astrocytes

**Figure 5:**
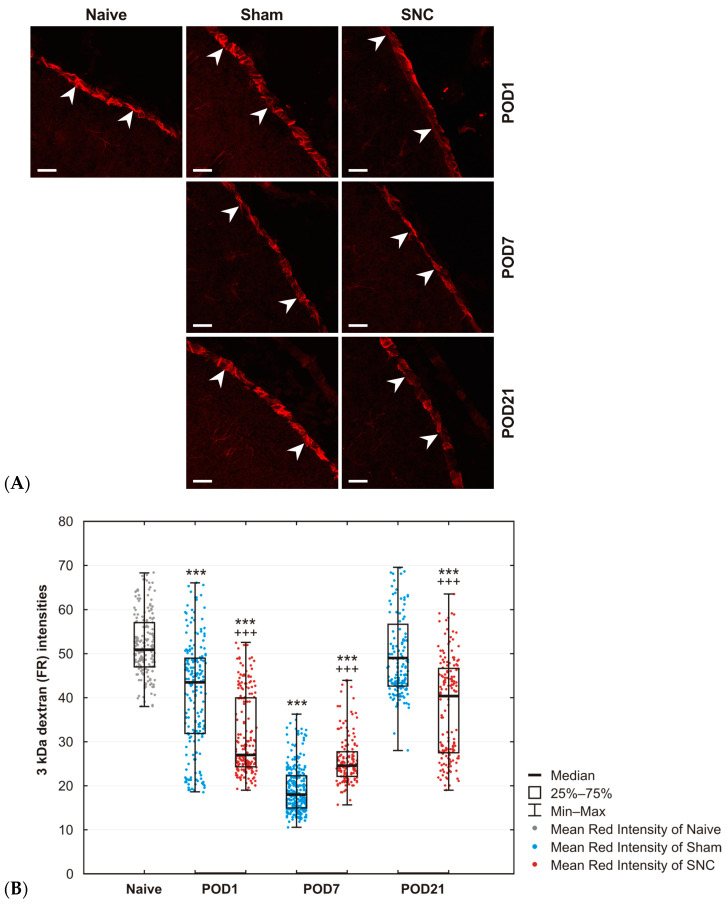
(**A**) Representative images showing 3 kDa dextran captured in the GLS of the mPFC on maximal projection images obtained from at least six Z-stack images. Note the difference between operated animals on POD7 compared to animals operated on POD21. Arrowheads indicate GLS position. Scale bars = 100 μm. (**B**) A significant reduction in 3 kDa FR intensity was observed at most time points for both sham- and SNC-operated animals compared to naïve animals. Values at POD21 for sham-operated animals returned to those of naïve animals. Results are expressed as the median, interquartile range (25th–75th percentile), and minimum–maximum values. *** *p* < 0.001 vs. naïve animals; +++ *p* < 0.001 compared to sham-operated animals, (n = 2, for each group). The dynamics of AQP4-IF intensities in subpial astrocytes following various survival periods after sham and SNC operations. *** *p* < 0.001 vs naïve animals; +++ *p* < 0.001 compared to SNC, (n = 3, for each group).

The results demonstrated a greater abundance of fixable TRITC-conjugated dextran 3 kDa (Fluoro-Ruby, FR) in subpial astrocytes of naïve control animals, indicating that this small water-soluble dextran was effectively taken up by the GLS barrier. However, both sham and SNC operations on POD1 and POD7 resulted in a significant reduction of FR levels in the GLS compared to those of naïve control animals. In contrast, the FR levels in the GLS of sham-operated rats on POD21 were comparable to those in the naïve GLS, indicating a recovery to baseline. Conversely, SNC on POD21 induced a modest increase in FR levels within subpial astrocytes that remained lower than those observed in naive rats (Figure 5A,B).

## 3. Discussion

To date, much attention has been paid to the reactivation of parenchymal astrocytes [10] and astrocyte endfeet of the GLP under various pathological conditions [9,18,31]. In addition, there is compelling evidence that peripheral nerve injury can cause the opening of both the BBB and the blood–cerebrospinal fluid barrier [19,22]. However, our understanding of the response of subpial astrocytes after nerve injury is limited, even though these astrocytes cover a large surface area of the CNS and provide a boundary for cellular and molecular exchange between the subpial space and the CNS parenchyma.

In our previous experiments, we observed the reactivation of subpial astrocytes through the alteration of glial fibrillary acidic protein (GFAP), glutamine synthetase (GS), and NFκBp65 immunofluorescence intensities after sham and SNC operations. Furthermore, reactive subpial astrocytes of the GLS exhibited more extensive cytoplasmic processes and formed a dense meshwork in cortical layer-I after both sham and SNC operations compared to the naive control [26].

### 3.1. Changes in Cx43 in Subpial Astrocytes

Astrocytes express high levels of Cx43 and other connexins, which are associated with gap junctions that connect the cytoplasm of neighboring cells and their cytoplasmic processes in a multicellular syncytium [32,33]. Thus, Cx43 is an important functional feature of astrocytes that is associated with their participation in the redistribution of water, ions, and small molecules up to a molecular mass of about 1 kDa [34]. It is well known that Cx43 is expressed in the GLP, indicating the presence of gap junctions between astrocyte endfeet [11,18]. However, the changes of Cx43 protein in the GLS after nerve injury have not been elucidated. We observed punctate Cx43-IF in subpial astrocytes detected by double immunostaining with GFAP antibody on the mPFC surface. In addition, Cx43-immunopositive dots were also observed in sections where the pia mater was present, consistent with the presence of gap junctions in this leptomeningeal layer [35]. Subpial astrocytes of the GLS covering the mPFC showed a statistically significant increase in integrated intensities of Cx43-IF after both sham and SNC operations for all survival times compared to the naïve control. The Cx43 protein levels increased to a peak on POD7, after which they subsequently declined.

Peripheral nerve injury induces various cellular and molecular changes in the mPFC that are related to the affective behavioral changes frequently associated with neuropathic pain [36,37,38]. Among these changes is the increased reactivity of parenchymal astrocytes in the spinal dorsal horn [39] and mPFC [10,40], accompanied by enhanced expression of Cx43 associated with inflammatory profiling of astrocytes [11,41]. The role of Cx43 alteration in the mPFC in behavioral changes was supported by treatment with Cx43 inhibitors, which resulted in the attenuation of the affective behavioral changes [42]. Currently, it is unknown if subpial astrocytes of the GLS have direct contact with mPFC neurons or form syncytial connections with parenchymal astrocytes. In contrast to reactive parenchymal astrocytes exhibiting long-lasting enhanced Cx43 [43,44], subpial astrocytes covering the mPFC display only transient upregulation of Cx43. It is also difficult to determine the contribution of Cx43 changes in subpial astrocytes covering the mPFC surface to depressive or other affective behavioral changes.

Our previous experiments demonstrated that both sham and SNC operations induced more extensive cytoplasmic processes in the cortical layer-I [26]. We can also expect a comparably increased number of horizontally oriented cytoplasmic processes in the GLS proper, although their evaluation is a methodological problem. Nevertheless, gap junctions between subpial astrocytes and their cytoplasmic processes have been reported [27]. Therefore, the increased levels of Cx43 demonstrated the multiplication of gap junctions between subpial astrocytes and their numerous cytoplasmic processes in the GLS induced by both sham and SNC operations. These changes in subpial astrocytes may be associated with enhanced horizontal signaling at the cortical surface after both sham and SNC operations. Moreover, gap junctions facilitate communication between astrocytes through the rapid exchange of ions and small molecules, including glutamate [45,46,47]. Intriguingly, both sham and SNC operations induced a significant decrease in glutamine synthetase (GS) levels during the same survival periods as the increase in Cx43, likely resulting in increased glutamate levels in the GLS [26]. However, the relevance of these observations requires further investigation and clarification. On the other hand, the dynamics of Cx43 regulation in subpial astrocytes following both sham and SNC operations might reflect their inflammatory profiling [48] as suggested by the timing coincidence with changes in phosphorylated NFκB [26] or with the development of inflammatory reactions and the opening of the blood–nerve barrier during Wallerian degeneration distal to nerve injury [49,50].

### 3.2. Changes in AQP4 in Subpial Astrocytes

AQP4 is a water channel that is widely expressed throughout the brain, particularly in the perivascular astrocyte endfeet of the GLP [5,51]. Although there is a difference in cellular composition between the rat GLS (astrocyte bodies and their processes) and the GLP (astrocytic endfeet), these structures share many similarities. In particular, astroglial AQP4 serves as a major molecular pathway for water and solute permeability in the brain and plays a critical role in maintaining water balance in the CNS parenchyma [52]. Immunopositive staining for AQP4 has been described in subpial astrocytes of naïve rats [28,29]. The critical role of AQP4 in subpial astrocytes for water content in the CNS parenchyma was confirmed using the AQP4 knockout (AQP4−/−) mouse with loss of AQP4 in subpial astrocytes [53], as well as by alterations in AQP4 immunostaining in the GLS covering the injured spinal cord [54].

Our semiquantitative image analysis revealed a significant reduction in AQP4-IF intensity, first observed after the sham operation on POD1 and then after both sham and SNC operations on POD3, POD7, and POD14. AQP4 protein returned to levels close to those measured in the GLS of naive rat controls after both sham and SNC operations on POD21. Therefore, the transiently reduced levels of AQP4 protein in subpial astrocytes suggest impaired transport of water and solutes between the CSF in the subpial space and the mPFC parenchyma, induced by sham and SNC operations during the period from POD3 to POD14. These changes in AQP4 levels induced by both sham and SNC operations likely reflect the reactivity of subpial astrocytes covering the mPFC in reaction to peripheral tissue and nerve injuries. Traumatic peripheral tissue injury like skin and muscle incisions in sham operations produces inflammatory mediators and their increase in blood circulation [55]. Wallerian degeneration distal to nerve injury is also considered to be a type of sterile inflammation [50]. These inflammatory reactions in both sciatic nerve injury and tissue injury alone initiate molecular signals that alter the function of astrocytes [56] and may modulate AQP4 protein levels in subpial astrocytes.

It is well-known that AQP4 is involved in the transport of both water and solutes and is investigated using water-soluble molecular tracers, such as labeled dextran of various molecular weights [9,15,57]. Fixable TRITC-conjugated dextran (FR) with a molecular weight of 3 kDa is widely used as a water-soluble tracer for studying brain barriers. In this study, 3 kDa FR was used to test the possible role of subpial astrocytes, which are located on the surface of cortical parenchyma and in contact with CSF in subpial space.

Fluorescence of 3 kDa FR was accumulated in subpial astrocytes of naïve rats and rats after both sham and SNC operations on POD1, POD7, and POD21. The question arises: what is the pathway for the penetration of intravenously administered 3 kDa FR to be taken up by the pia mater and subpial astrocytes at the cortical surface? Heavy loading of subpial astrocytes from naïve rats suggests that there is a pathway for partial penetration of 3 kDa FR from the blood into the CSF without injury. The likely pathway for penetration is an incomplete blood–CSF barrier that allows at least partial penetration of 3 kDa FR in the choroid plexus from blood plasma into the CSF [58]. This is possible through transcellular pathways [59], but paracellular transport through tight junctions of cuboidal cells cannot be excluded [60,61]. Additionally, a peripheral nerve injury induces cellular changes in the choroid plexus that may be associated with the attenuation of the blood–CSF barrier [22], facilitating the penetration of 3 kDa FR from the blood to the CSF. A peripheral nerve injury nerve may also weaken the BBB [19], thereby allowing the diffusion of 3 kDa FR from the blood into the perivascular space, which directly communicates with the CSF in the subpial space [14].

The CSF containing FR can reach the cortical surface covered by the pia mater and subpial astrocytes of the GLS. The portion of 3 kDa FR not captured by the pia mater is present in the CSF, filling the subpial space and coming into contact with subpial astrocytes. Our results demonstrated that the rat subpial astrocytes can effectively take up 3 kDa FR present in the CSF of the subpial space. This highlights the role of the GLS formed by subpial astrocytes as a structural barrier for balancing solutes in the CSF and cortical parenchyma under normal conditions. However, both sham and SNC operations induced alterations in subpial astrocytes, which were associated with changes in the uptake of FR. After slow intravenous injection, we observed a decrease in the uptake of 3 kDa FR in the GLS of sham- or SNC-operated rats compared to that of naïve rats. This decrease in FR uptake coincided temporally only at POD7 with the decrease in AQP4 protein levels in subpial astrocytes. Although AQP4 is currently known to play a role in regulating water and solute trafficking within astrocytes [30], further experiments are needed to demonstrate the correlation between AQP4 and solute uptake in subpial astrocytes.

A mutual relationship between Cx43 and AQP4 proteins has been documented in experiments with the GLP [62,63]. However, changes in AQP4 and Cx43 proteins in subpial astrocytes of the GLS occurred at different times after sham and SNC operations and thus did not correlate well.

### 3.3. Changes in Cx43 and AQP4 in Subpial Astrocytes after Both Sham and SNC Operations

Finally, our results demonstrated changes in Cx43 and AQP4 proteins induced in subpial astrocytes not only by a nerve lesion but also after a tissue injury during a sham operation. Numerous published results have shown that a peripheral nerve injury or associated inflammatory conditions lead to significantly greater reactivity and inflammatory profiling of parenchymal astrocytes in various cortical regions compared to sham operation [40,64]. This reactivity of parenchymal astrocytes in neuronal pathways is primarily caused by changes in neuronal activities induced by injury to peripheral axons [21,65,66]. In contrast, our results from Cx43 and AQP4 analyses suggest that changes in subpial astrocytes on the mPFC surface are likely induced by molecular cues released into the circulation from injured peripheral tissue or nerve. Such molecular cues can be encapsulated in small extracellular vesicles (sEVs), which are released by cells of injured tissues, including peripheral nerves [67,68,69,70]. Some proteins found in sEVs were unique to sham control surgery, suggesting that sham surgery itself can induce molecular changes in remote structures [67]. In addition, a large number of miRNAs were found in sEVs from both sham- and spared nerve injury-operated mice [68].

In recent years, accumulating evidence has suggested the essential role of non-coding RNAs, including miRNAs, in the regulation of Cx43 and AQP4 in experimental models based on peripheral nerve injury [71,72]. For example, miR-224 and miR-19a target not only AQP4 but also Cx43 proteins, suggesting their important roles in the regulation of astrocyte connectivity and water permeability [73]. In addition, miR-128-3p is known to be involved in the regulation of AQP4 protein levels [74], while miR-23b-3p and miR-1 are involved in the regulation of Cx43 in the nervous system [75,76]. These molecular cues in sEVs are transported via the blood to the choroid plexus, where they may diffuse across the attenuated blood–cerebrospinal fluid barrier into the CSF [22,77], subsequently activating subpial astrocytes. Different signal molecules in circulating sEVs may induce much more pronounced molecular changes in remote structures after sham operation than following nerve injury [68], similar to some changes observed in Cx43 and AQP4 protein levels in subpial astrocytes.

## 4. Materials and Methods

### 4.1. Animals and Surgical Procedures

Experiments were performed on 47 adult male rats (Wistar, 200–250 g). The rats were maintained at 22–24 °C with a 12 h light/dark cycle under specific pathogen-free conditions in the animal housing facility of Masaryk University. Sterilized food and water were available ad libitum. All surgical procedures were performed under aseptic conditions by the same person in accordance with the European Convention for the Protection of Vertebrate Animals used for Experimental and Other Scientific Purposes, and the protocol was approved by the Expert Committee for the Protection of Laboratory Animals of the Faculty of Medicine, Brno, Czech Republic.

Animals were anesthetized by intraperitoneal injection (i.p.) of a mixture of ketamine (60 mg/kg) and xylazine (7.5 mg/kg), and the left sciatic nerve was exposed in mid-thigh by blunt dissection. In the sciatic nerve compression (SNC) group of animals (n = 15), three ligatures (3–0 Ethicon) were tied around the nerve at 1 mm intervals to reduce the nerve diameter by approximately one-third of its original diameter. The sciatic nerve of sham-operated rats (n = 15) was only exposed without any injury. Muscles and skin were closed with sutures, and animals were allowed to survive until postoperative day (POD) 1, 3, 7, 14, and 21 (n = 3 for each group). Three intact rats were used as naïve controls.

For the experiments involving the uptake of 3 kDa dextran, an additional 6 sham-operated rats and 6 SNC-operated rats were allowed to survive on POD1, 7, and 21 (n = 2 for each group). Additionally, 2 rats without any surgical treatment were used as naïve controls.

No analgesia was used, as this would have interfered with the purpose of the study. No animals were excluded from the experimental group sets for the following evaluation.

### 4.2. Immunohistochemical Staining

Animals were deeply anesthetized with a lethal dose of sodium pentobarbital (80 mg/kg body weight, i.p.) and perfused transcardially with 100 mL phosphate-buffered saline (PBS, 10 mM sodium phosphate buffer, pH 7.4, containing 0.15 M NaCl) followed by 500 mL of Zamboni’s fixative [78]. Brains were removed and immersed in Zamboni’s fixative overnight at 4 °C. Removed brains were washed in 10% and 20% phosphate-buffered sucrose for 24 h. A thick coronal slice of the mPFC, oriented nearly perpendicular to the cortical surface, was carefully cut from each brain at a position between 3.7 and 1.5 mm from Bregma [79] using manual microdissection under a stereomicroscope. The slices were placed on the cryostat holder, blocked in Tissue-Teck OCT compound (Miles, Elkhart, IN, USA), and serial cryostat sections (12 µm) were cut (Leica 1800 cryostat; Leica Microsystems, Wetzlar, Germany).

Sections were mounted on chrome–alum-covered slides, air-dried, and processed for immunohistochemical staining for Cx43 and AQP4 under the same conditions. Briefly, mPFC sections from naive, sham-, and SNC-operated rats were simultaneously immunostained with primary antibodies and visualized with corresponding secondary antibodies (Table 1). To detect cell nuclei, the sections were stained with Hoechst 33342 and mounted in an aqueous mounting medium (Vectashield, Vector Laboratories, Newark, CA 94560, USA). Control sections were incubated either without the primary antibodies or by replacing the primary antibodies with the IgG isotype. The control sections did not show any immunostaining.

### 4.3. Double Immunohistochemical Staining

To demonstrate Cx43 and AQP4 immunostaining in subpial astrocytes, the mPFC sections were double immunostained with mouse monoclonal anti-Cx43 or rabbit polyclonal anti-AQP4 antibody simultaneously with rabbit polyclonal anti-GFAP or chicken polyclonal anti-GFAP antibody, respectively. Briefly, the sections were incubated with mouse monoclonal anti-Cx43 (1:100) for 240 min, followed by AlexaFluor647-conjugated goat anti-mouse secondary antibody for 90 min. After thorough rinsing, the same sections were incubated with rabbit anti-GFAP for 180 min, followed by FITC-conjugated goat anti-rabbit secondary antibody for 90 min.

To detect AQP4 in subpial astrocytes, the mPFC sections were incubated with rabbit polyclonal antibody against AQP4 overnight at room temperature, followed by incubation with Alexa Fluor 549-conjugated goat anti-rabbit secondary antibody for 90 min. After thorough rinsing, the sections were then incubated with chicken anti-GFAP antibody for 180 min, and immunostaining was visualized by incubation with AlexaFluor488-conjugated goat anti-chicken secondary antibody for 90 min.

Control sections were incubated without primary antibodies or with a reverse combination of primary and secondary antibodies. No immunostaining was observed in the control sections.

### 4.4. Uptake of 3 kDa Dextran by Subpial Astrocytes

To investigate the role of subpial astrocytes in the uptake of molecules, fixable TRITC-conjugated dextran (Fluoro-Ruby, 3 kDa; Molecular Probes, Eugene, OR, USA) was used. Fluoro-Ruby (FR) was dissolved at a concentration of 10 mg/mL in *aqua pro injectione*, stored as frozen aliquots at −70 °C, thawed immediately before injection, and diluted with artificial cerebrospinal fluid (ACSF; [80]) to a concentration of 1 mg/100 µL before use.

FR solution was slowly injected (50 μL at 10 μL/min) into the jugular vein under deep anesthesia of naïve rats (n = 2) and rats after both sham and SNC operations on POD1, POD7, and POD21. One hour after injection, the deeply anesthetized rats were transcardially perfused with Zamboni fixative solution, and the brains were then removed and postfixed in the same fixative solution for 24 h. Coronal cryostat sections of mPFC (12 µm) were cut, stained with Hoechst 33342, and mounted in an aqueous mounting medium (Vectashield, Vector Laboratories, Newark, NJ, USA).

### 4.5. Microscopy and Image Analysis

The immunostained sections were observed and analyzed by blinded persons with knowledge of the experimental groups using an epifluorescence microscope (Nikon Eclipse) equipped with a Nikon DS-Ri1 camera (Nikon, Prague, Czech Republic) and a stabilized power supply for the lamp housing. In addition, double immunostaining was verified by analysis using a Leica TCS SP5 confocal microscope (Leica Microsystems, Wetzlar, Germany) with a 20× oil immersion objective. Only sections containing a continuous and distinct layer of subpial astrocytes were used for analysis.

#### 4.5.1. Semiquantitative Analysis of Cx43

Images of GFAP and Cx43 double-immunostained mPFC sections were obtained using a Z-stack mode with a Leica TCS SP5 confocal microscope at 20× magnification (20× oil immersion objective). Six Z-stack images with 1.6 μm steps were acquired from each specimen, and a maximum projection image combining all the sequential images was processed and saved in TIFF format containing the red (Cx43) and green (GFAP) channels. For quantitative image processing, gray-scale images were converted from the R and G channels after background subtraction such that pixels with a value of 0 represented complete negative staining and pixels with a value of 255 represented complete saturation. The binary mask indicating the position of the GLS was obtained by thresholding from the G channel and after manual editing. The integrated intensity of all pixels corresponding to Cx43 immunofluorescence was measured below the binary mask using the NIS-Elements image analysis system (Nikon, Prague, Czech Republic) and recalculated to the area of the GLS profile (3000 µm^2^) for individual images.

#### 4.5.2. Semiquantitative Analysis of AQP4

For semiquantitative analysis, the intensities of AQP4 immunofluorescence (AQP4-IF) labeling in the GLS were measured using the image analysis system (NIS-Elements image analysis system) according to our previously published protocols [26,81]. Briefly, the AQP4-immunostained GLS was detected for measurement by a thresholding technique after background subtraction, transformed to binary mode, and the binary masks were manually edited as needed. The limitation of the GLS was also checked by a line of cell nuclei stained with Hoechst. At least 6 profiles of the GLS were measured for each group of animals. Immunofluorescence intensities were expressed as mean ± S.D.

#### 4.5.3. Analysis of the 3 kDa Dextran Taken up by Subpial Astrocytes

Z-stack images were acquired for each mPFC section using a Leica TCS SP5 confocal microscope at 20× magnification (20× oil immersion objective), with 8 to 10 1.6 μm steps. The maximum projection image of all the sequential steps was processed and saved in TIFF format, containing the red (FR) channel. The binary mask indicating the GLS position was prepared by thresholding and manually edited if necessary. The mean intensity of the red channel was measured using the NIS-Elements image analysis system under the binary mask on at least 6 GLS profiles for each experimental group and expressed as mean ± S.D.

### 4.6. Statistical Analysis

Statistical differences between naïve and sham-operated or sham- and SNC-operated animals for each survival period were tested by the Mann–Whitney test using a STATISTICA 12 (StatSoft, Inc., Tulsa, OK, USA). Results of Cx43 and AQP4 protein levels are expressed as mean ± SD, while results of 3 kDa FR uptake measurement are expressed as the median, interquartile range (25th–75th percentile), and minimum–maximum values. A *p*-value marked with * is used to describe the statistical difference between naïve and operated animals, *** *p* < 0.001; * *p* < 0.05, while + is used to describe the statistical difference between sham- and SNC-operated animals, +++ *p* < 0.001, ++ *p* < 0.005, + *p* < 0.05.

## Figures and Tables

**Table 1:** List of primary and secondary antibodies used for immunofluorescence detection.

Protein	Antibody	Source	Product	City, Country	Cat. No	Dilution
Cx43	mAb *	mouse	Millipore	Burlington, USA	MAV3067	1:100
AQP4	pAb *	rabbit	Cell Signaling	Danvers, USA	59678	1:800
GFAP	pAb *	rabbit	Dako	Santa Clara, USA	Z 0334	1:250
GFAP	pAb *	chicken	Abcam	Cambridge, UK	ab4674	1:500
**Secondary antibody**						
Anti-mouse-A647	pAb *	goat	Jackson	Ely, UK	115-605-146	1:100
Anti-rabbit-A549	pAb *	goat	Abcam	Cambridge, UK	ab96900	1:100
Anti-rabbit-FITC	pAb *	goat	Jackson	Ely, UK	111-095-144	1:100
Anti-chicken-A488	pAb *	goat	Abcam	Cambridge, UK	ab150173	1:500

* pAb, polyclonal antibody; mAb, monoclonal antibody.

## Data Availability

The original contributions presented in the study are included in the article; further inquiries can be directed to the corresponding author.
